# Biomechanical and clinical comparison of different prosthetic in reconstruction following total *en bloc* spondylectomy in the thoracolumbar spine: based on finite element analysis and clinical data 

**DOI:** 10.3389/fbioe.2025.1573086

**Published:** 2025-07-01

**Authors:** Zhangui Gu, Long Ma, Qiang Liu, Kun Wang, Zongqiang Yang, Ningkui Niu, Jiandang Shi

**Affiliations:** ^1^ Department of Orthopedic, General Hospital of Ningxia Medical University, Yinchuan, China; ^2^ First Clinical Medical College of Ningxia Medical University, Yinchuan, China

**Keywords:** total *en bloc* spondylectomy, artificial vertebral bodies, titanium mesh cage, finite element analysis, clinical research

## Abstract

**Objectives:**

To analyze and compare the biomechanical differences and clinical efficacy of artificial vertebral bodies (AVBs) versus traditional titanium mesh cages (TMCs) reconstruction following total *en bloc* spondylectomy (TES).

**Materials and Methods:**

Finite Element Analysis: A finite element model of T12-L5 vertebrae from a healthy adult was utilized to construct two reconstruction models following L2 TES: Group A (AVB) and Group B (TMC). Using ANSYS software, flexion-extension, lateral bending, and axial rotation loading conditions were simulated to comparatively analyze stress distribution at the prosthesis-endplate interface and biomechanical characteristics of the fixation system; Clinical research: This retrospective study included 20 thoracolumbar tumor patients who underwent posterior TES at our institution from January 2014 to October 2024, divided into AVBs (*n* = 10) and TMCs (*n* = 10) reconstruction groups. Systematic comparisons were performed for perioperative parameters (operative time, blood loss, hospital stay), with dynamic assessments of preoperative to final follow-up Visual Analog Scale (VAS) pain scores, American Spinal Injury Association (ASIA) neurological classifications, and Karnofsky Performance Status (KPS) scores. Radiographic measurements of vertebral height and angular alignment changes were conducted to comprehensively evaluate reconstruction outcomes.

**Results:**

Finite element analysis revealed that the TMC model exhibited significant stress concentration phenomena across all motion modes compared to the AVB model. Specifically, the stress on the L1 inferior endplate was 50.09%, 17.48%, 74.07%, 133.83%, and 87.23% higher during extension, left lateral bending, right lateral bending, left axial rotation, and right axial rotation, respectively. The L3 superior endplate demonstrated similar stress patterns but with smaller magnitudes. In both implant models, peak stresses occurred during extension and axial rotation, followed by lateral bending, with minimal stress observed during flexion. For the posterior fixation system, no significant differences in maximum stress were observed between the two prosthetic configurations; Clinically, Group A demonstrated significantly lower implant subsidence rates (10% vs 70%) and superior outcomes in intervertebral height loss (1.33 ± 0.82 mm vs 12.36 ± 7.79 mm) and angular loss (*p* < 0.05). No statistically significant differences were identified between groups regarding hospitalization duration, operative time, intraoperative blood loss, VAS scores, KPS scores, or ASIA grade improvements (*p* > 0.05).

**Conclusion:**

Following TES, the AVB demonstrated more uniform stress distribution and superior biomechanical performance compared to the TMC. Additionally, the AVB effectively reduced implant subsidence rates, maintained intervertebral height, corrected kyphotic deformities, and exhibited enhanced biomechanical stability and clinical efficacy.

## 1 Introduction

Total *en bloc* spondylectomy (TES) represents a complex and challenging surgical procedure typically indicated for patients with severe vertebral destruction caused by spinal tumors, severe trauma, or infections. Postoperative spinal reconstruction aims to restore spinal stability, maintain anatomical alignment, and promote functional recovery (Jones et al., 2018). Biomechanical studies (Disch et al., 2008; Wang X. et al., 2021; Kang et al., 2024), have demonstrated that TES requires posterior multisegment pedicle screw fixation combined with anterior vertebral body reconstruction techniques to provide adequate stability and restore spinal biomechanical properties. In current clinical practice, artificial vertebral bodies (AVBs) and conventional titanium mesh cages (TMCs) are the two primary spinal reconstruction options. Although both techniques aim to achieve similar clinical outcomes, significant differences may exist in terms of biomechanical performance and long-term efficacy.

Currently, TMCs have gained widespread clinical adoption due to their procedural simplicity, broad applicability, and cost-effectiveness. However, concerns persist regarding their insufficient long-term stability and adaptability in spinal reconstruction (Cheng et al., 2022; Chen et al., 2023; Dong et al., 2024). In recent years, AVBs have attracted increasing attention for their enhanced capacity to mimic the natural anatomical structure of vertebral bodies, highlighting their advantages in providing superior structural support and potentially reducing risks of implant subsidence or failure, as evidenced by emerging reports (Zhou et al., 2022; Hu et al., 2023). Nevertheless, comparative biomechanical analyses between AVBs and TMCs remain insufficiently explored in the literature, and investigations integrating clinical outcome data are particularly scarce.

The objective of this study is to compare the biomechanical differences and clinical efficacy between artificial vertebral body implantation and traditional titanium mesh cage implantation following TES through finite element analysis combined with a retrospective review of clinical cases. We will systematically assess the mechanical properties, postoperative complication rates, and long-term clinical outcomes of both approaches, aiming to provide a more evidence-based scientific foundation for clinical decision-making in spinal reconstruction surgery.

## 2 Materials and methods

### 2.1 Finite element analysis

#### 2.1.1 Intact thoracolumbar finite element model

To establish the finite element model, we selected a 26-year-old healthy male volunteer (height: 175 cm, weight: 70 kg) with no history of spinal diseases, trauma, or surgical interventions. Routine thoracolumbar radiographic examination revealed no significant degenerative changes. CT data scans of the thoracolumbar segment were performed on volunteers using a 64-slice spiral computed tomography scanner (Philips, Amsterdam, Netherlands) with a slice interval of 0.625 mm. Image datasets encompassing six vertebral bodies (T12-L5) and five intervertebral discs were acquired and exported in Digital Imaging and Communications in Medicine (DICOM) format. Subsequent three-dimensional reconstruction was conducted using Mimics 21.0 (Materialise NV, Leuven, Belgium) to precisely delineate vertebral anatomy and generate surface-rendered 3D models spanning T12-L5, which were ultimately saved in Standard Tessellation Language (STL) format files.

The STL files were imported into Geomagic 2021 software (3D Systems, North Carolina, USA) for refinement, including smoothing, noise reduction, and surface fitting to achieve smooth surfaces meeting analytical requirements, generating STEP-format files. These files were then assembled in SOLIDWORKS 2022 (Dassault Systèmes, France) for tissue reconstruction and modeling of AVB and TMC, ultimately establishing three models: an intact T12-L5 model, a post-L2 corpectomy artificial vertebral body reconstruction model, and a titanium mesh cage reconstruction model, all exported as Parasolid (.x_t) files. Finally, the models were imported into ANSYS (ANSYS, Inc., USA) for material assignment, meshing, application of boundary conditions/loads, and stress distribution analysis.

**FIGURE 1:**
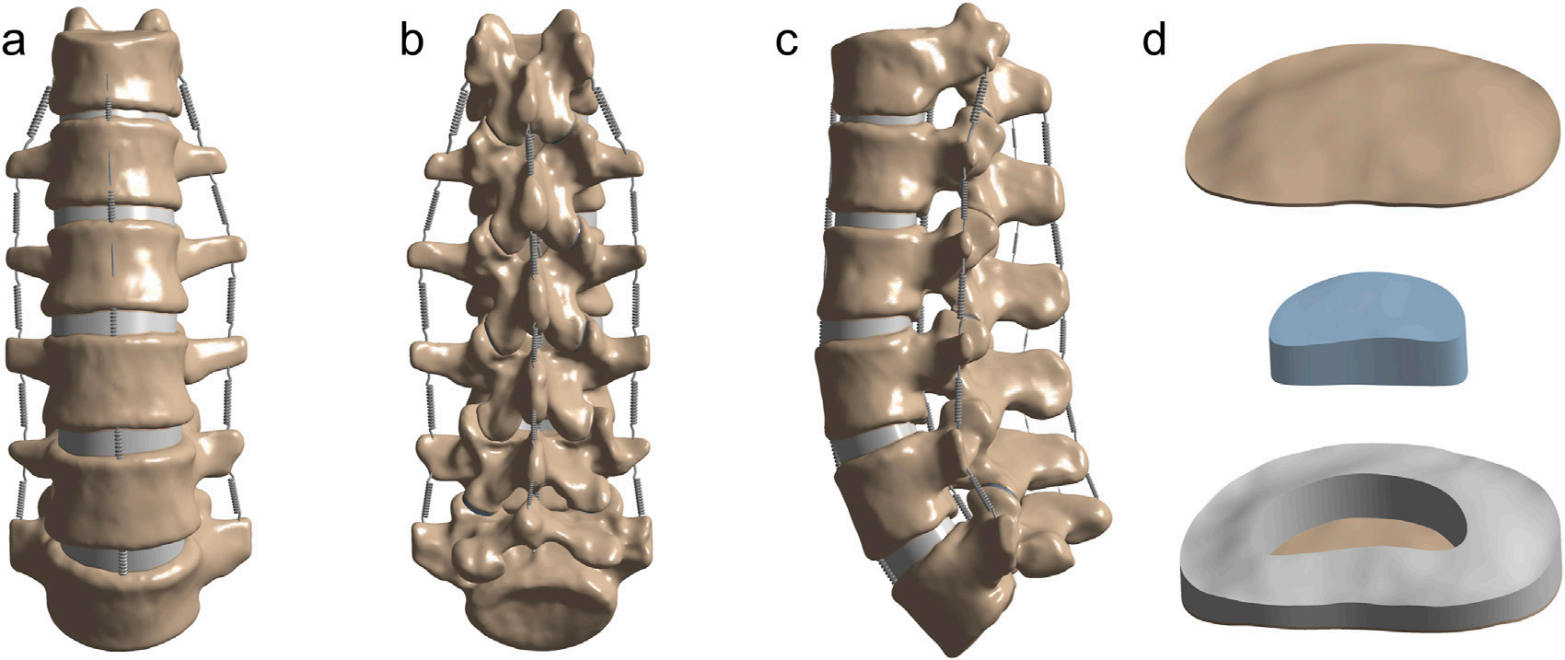
Anatomical configuration of the thoracolumbar finite element model **(A–C)** Front, back and side views of the thoracolumbar finite element model. **(D)** Bony endplates and intervertebral discs (including nucleus pulposus, annulus fibrosus).

The intact T12-L5 finite element model is shown in Figures 1A–D. The cortical bone, articular cartilage, and bony endplates were assigned thicknesses of 1.5 mm, 0.3 mm, and 0.5 mm, respectively. Intervertebral discs were differentiated into nucleus pulposus and annulus fibrosus components, with the nucleus pulposus occupying 30%–40% of the disc volume, as illustrated in Figure 1D. Spring elements were implemented to represent seven key spinal ligaments: the anterior longitudinal ligament (ALL), posterior longitudinal ligament (PLL), ligamentum flavum (LF), intertransverse ligaments (ITL, bilateral), interspinous ligament (ISL), and supraspinous ligament (SSL). The properties of the ligaments were set by stiffness. The contact relationships between the disc and vertebral bodies, vertebral bodies, ligaments, and between the disc and ligaments are all set as “binding”. An element size of 2.0 mm was selected to balance computational accuracy and load-bearing requirements. Under these meshing parameters, the complete T12-L5 finite element model comprised 948,525 discrete elements.

#### 2.1.2 Validation of the finite element model

The spinal motions in the sagittal, coronal, and transverse planes were defined as flexion-extension, lateral bending, and axial rotation, respectively. The inferior surface of the L5 vertebra was fully constrained, while a 7.5 N·m pure moment combined with a 200 N axial load was applied to the superior surface of T12. The range of motion (ROM) was measured and compared with previously reported experimental data (Schmoelz et al., 2010; Alizadeh et al., 2013; Wang et al., 2019).

#### 2.1.3 Finite element postoperative model

Finite element models of pedicle screws (6.5 × 45 mm), rods (5.5 mm diameter), titanium mesh cage (TMC), and artificial vertebral body (AVB) (Johnson & Johnson) were developed using SOLIDWORKS software (Dassault Systèmes, Paris, France). The TMC and AVB dimensions measured 17 × 22 mm and 18 × 22 mm, respectively, with both constructs fabricated from medical-grade titanium alloy (Ti-6Al-4V). The material properties used in the finite element model (Table 1) were derived from previous reports (Bonnheim and Keaveny, 2020; Liang et al., 2020).

**TABLE 1:** Material parameters of different materials in the finite element model.

Structure	Young’s modulus (MPa)	Poisson’s ratio
Cancellous bone	100	0.3
Cortical bone	12,000	0.3
Cartilage	10	0.4
Annulus fibrosus	4.2	0.3
Nucleus pulposus	1	0.499
Endplate	1000	0.4
Screw-rod system	110,000	0.3
Mesh cage		
Artificial vertebra		
Bone graft	100	0.3

**FIGURE 2:**
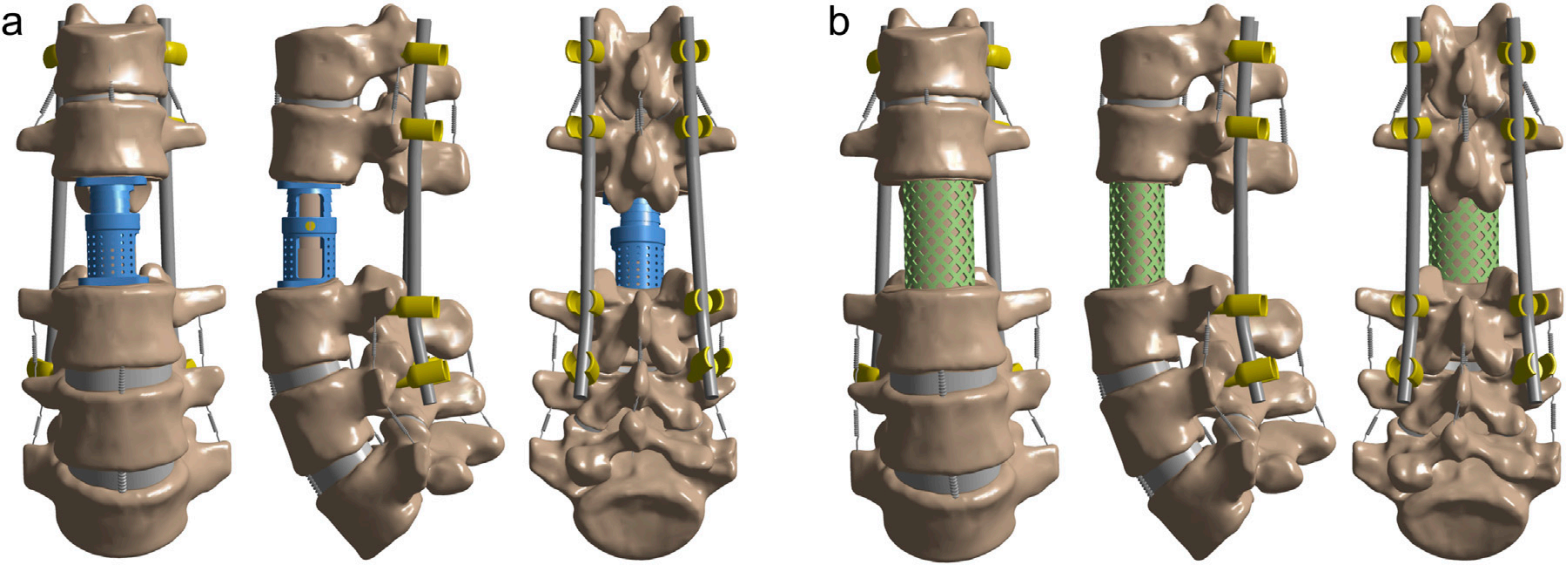
Post-TES reconstruction models of L2. **(A)** Model of artificial vertebral body (AVB) reconstruction after L2 TES; **(B)** Model of titanium mesh cage (TMC) reconstruction after L2 TES.

Figure 2 illustrates two postoperative finite element models following L2 TES. The surgical simulation included complete resection of the L2 vertebral body, L1-L2 and L2-L3 intervertebral discs, and associated anterior/posterior longitudinal ligaments. Posterior stabilization was achieved through bilateral pedicle screw fixation spanning two levels above and below the affected vertebra (T12/L1 and L3/L4). Defect reconstruction employed either: (A) an artificial vertebral body or (B) a titanium mesh cage, both filled with cancellous bone graft material (material properties detailed in Table 1). Eight 6.5 × 45 mm pedicle screws were implanted at T12, L1, L3, and L4 vertebral levels.

#### 2.1.4 Finite element simulation analysis

ANSYS 2022 (ANSYS, Inc., USA) was used to simulate spinal motion under defined boundary and loading conditions. The L5 vertebra was assumed fully constrained by applying fixed displacement and rotational constraints to its inferior endplate. Spinal motions in the sagittal, coronal, and transverse planes were respectively defined as flexion-extension, lateral bending, and axial rotation. Based on the capacity of the human body and previously published reference (Alizadeh et al., 2013), a 200 N axial load superimposed with a 7.5 N·m pure moment was applied to the superior endplate of the T12 vertebra to simulate multidirectional spinal movements (flexion-extension, lateral bending, and axial rotation).

### 2.2 Clinical research

#### 2.2.1 Object of study

A retrospective analysis was conducted on patients with thoracolumbar tumors who underwent total *en bloc* spondylectomy (TES) at the General Hospital of Ningxia Medical University from January 2014 to October 2024. The cohort was divided into two groups according to intraoperative vertebral reconstruction methods: Group A (artificial vertebral body reconstruction) and Group B (titanium mesh cage reconstruction). Inclusion Criteria: (1) Diagnosis of spinal tumors or other benign lesions requiring total *en bloc* spondylectomy (TES); (2) Presence of significant spinal cord or nerve compression symptoms with progressive worsening; (3) Good general condition, single vertebral involvement, and clear surgical indications; (4) Complete follow-up data. Exclusion Criteria: (1) Short life expectancy (<6 months); (2) Concurrent multiple organ metastases; (3) Recurrent tumor lesions; (4) Lost to follow-up or missing key data.

#### 2.2.2 Preoperative preparation

To address the potential risk of significant intraoperative bleeding, three patients in Group A (1 hemangioma, 1 giant cell tumor of bone, and 1 osteosarcoma) and four patients in Group B (2 hemangiomas, 1 aneurysmal bone cyst, and 1 myeloma) underwent preoperative transcatheter arterial embolization to reduce tumor vascularity and minimize blood loss. Additionally, all patients received standardized preoperative evaluations, including complete blood counts, coagulation profiles, cardiopulmonary function assessments, spinal imaging (CT and MRI), and tumor-related marker analyses (e.g., bone scintigraphy, tumor biomarkers). For patients requiring intraoperative transfusion, blood products were preoperatively prepared, and individualized surgical/perioperative management plans were developed based on clinical status. No adjunctive interventions affecting intraoperative bleeding, such as preoperative radiotherapy or specific pharmacological therapies were implemented.

#### 2.2.3 Operative procedures

Both patient groups underwent single-stage posterior TES. Under general anesthesia, the posterior approach was systematically performed to expose the pathological vertebrae and surrounding tissues, achieving complete resection of lesions and involved vertebral bodies while preserving the spinal cord and adjacent critical structures. Vertebral reconstruction in Group A was accomplished using AVBs, whereas Group B received TMCs for defect restoration. Both groups underwent posterior pedicle screw-rod instrumentation to restore and maintain spinal stability. All surgical procedures were guided by real-time intraoperative C-arm fluoroscopy to ensure precision in vertebral resection and reconstruction.

#### 2.2.4 Postoperative management

Postoperatively, patients received standardized supportive care including fluid resuscitation to maintain hemodynamic stability, combined analgesic and neurotrophic medications to facilitate recovery, and prophylactic broad-spectrum antibiotics based on intraoperative/postoperative infection risks. Drainage volume and fluid characteristics were closely monitored, with drains removed if output fell below 50 mL within 24 h and showed no abnormal features (e.g., hematogenic or purulent discharge), followed by continued wound observation. Mandatory bed rest was enforced for 2 weeks to reduce spinal loading and promote wound healing. After this period, patients initiated gradual ambulation under thoracolumbar brace protection, with activity intensity progressively adjusted according to radiographic follow-up and clinical recovery status. All patients were instructed to adhere to structured rehabilitation protocols for accelerated recovery.

#### 2.2.5 Postoperative follow-up and observation parameters

(1) Length of hospital stay, operative duration, and intraoperative blood loss; (2) Pain intensity assessed preoperatively and at immediate postoperative, 1-month, 3-month, and final follow-up intervals using the Visual Analogue Scale (VAS); (3) Neurological function evaluated preoperatively, immediately postoperatively, at 3-month, and final follow-up via the American Spinal Injury Association (ASIA) Impairment Scale; (4) Quality of life (QOL) quantified preoperatively, immediately postoperatively, 1-month, 3-month, and final follow-up using the Karnofsky Performance Status Scale(KPS); (5) Segmental height measured preoperatively, 7 days postoperatively, and at final follow-up. This parameter was defined radiographically as the distance between the midpoints of the inferior endplate of the superior adjacent vertebra and the superior endplate of the inferior adjacent vertebra on lateral X-rays. Height loss ≥3 mm was classified as implant subsidence, with magnitude and subsidence rate calculated; (6) Segmental angle measured postoperatively, 7 days postoperatively, and at final follow-up, determined by the angular divergence between the inferior endplate of the superior adjacent vertebra and the superior endplate of the inferior adjacent vertebra. Angular loss magnitude was computed.

#### 2.2.6 Statistical method

All statistical analyses were performed using IBM SPSS 28.0 software. Normally distributed continuous variables were described as mean ± standard ( $\bar{x}\pm s$ ) deviation, with inter-group comparisons conducted via independent samples T-test and intra-group comparisons via paired T-test. Non-normally distributed continuous variables were expressed as median (interquartile range), and inter-group comparisons employed the Mann-Whitney U test. Categorical variables were reported as frequency (%), with group comparisons analyzed by chi-square test or continuity-corrected chi-square test as appropriate. The significance level (α) was set at 0.05, with *p* < 0.05 considered statistically significant.

## 3 Results

### 3.1 Finite element analysis

#### 3.1.1 Model validation

**FIGURE 3:**
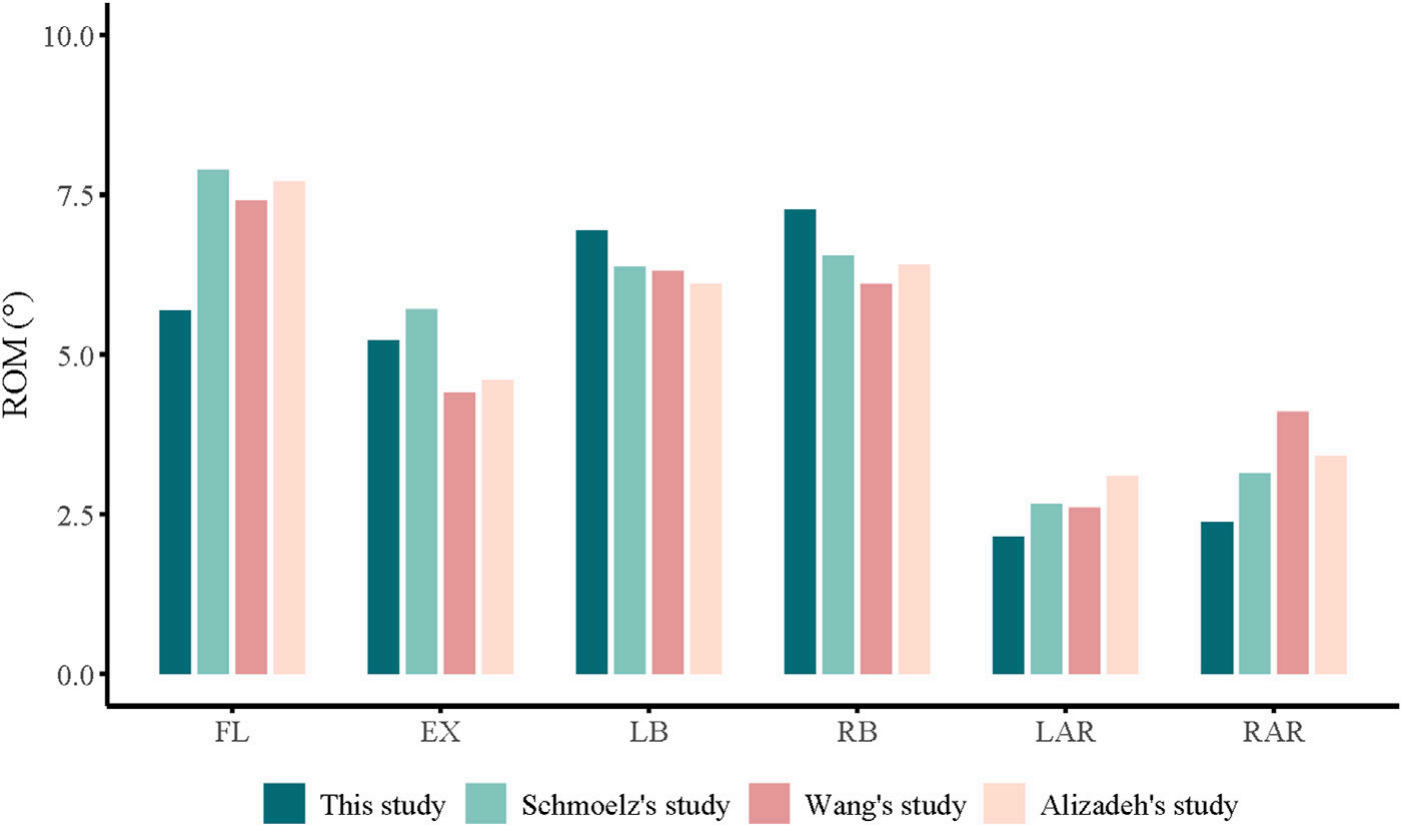
ROM of the T12-L5 in intact finite element model made in this study is compared with previously reported data.

The ROM measurements for flexion, extension, lateral bending, and axial rotation of the intact thoracolumbar model (T12-L2) were compared with findings from previous biomechanical studies and finite element models (Schmoelz et al., 2010; Alizadeh et al., 2013; Wang et al., 2019) (Figure 3). Minor discrepancies may stem from unavoidable factors such as individual variations, parameter configurations, and software processing. Overall, the model data exhibited strong concordance with published literature, indicating high precision of the established finite element model and validating its efficacy, thereby demonstrating its suitability for subsequent investigations.

#### 3.1.2 Von Mises stress distribution on prosthesis-adjacent endplates under different prosthetic supports

**FIGURE 4:**
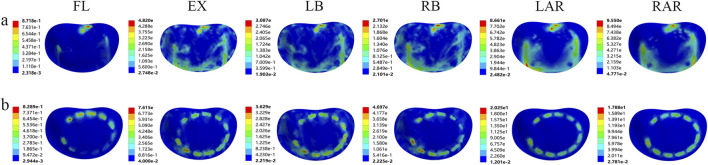
Von Mises stress of the lower endplate of L1 supported by two kinds of prostheses: **(A)** artificial vertebral body (AVB); **(B)** titanium mesh cage (TMC) (FL: flexion, EX: extension, LB: left lateral bending, RB: right lateral bending, LAR: left axial rotation, RAR: right axial rotation).

**FIGURE 5:**
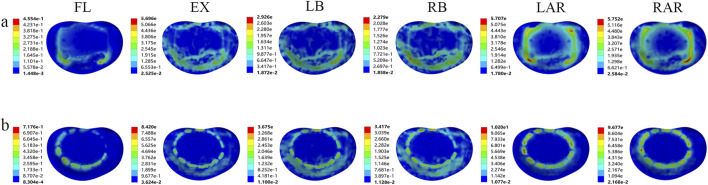
Von Mises stress of the upper endplate of L3 supported by two kinds of prostheses: **(A)** artificial vertebral body (AVB); **(B)** titanium mesh cage (TMC) (FL: flexion, EX: extension, LB: left lateral bending, RB: right lateral bending, LAR: left axial rotation, RAR: right axial rotation).

In both fixation models, the Von Mises stress results at prosthesis-adjacent endplates revealed that the stress values at the inferior endplate of L1 were nearly universally higher than those at the superior endplate of L3, except during flexion where comparable magnitudes were observed. During extension, the stress at the L1 inferior endplate in the TMC model was 50.09% higher than in the AVB model. Similar disparities persisted during left lateral bending (17.48% higher), right lateral bending (74.07%), left axial rotation (133.83%), and right axial rotation (87.23%). Additionally, pronounced stress concentration phenomena were identified in the TMC model across all motion modes (Figure 4). While the L3 superior endplate exhibited analogous trends, the stress variations were less pronounced (Figure 5). These findings indicate that the TMC model may impose greater biomechanical loading on endplates under specific kinematic conditions.

#### 3.1.3 Von mises stress distribution in different prostheses

**FIGURE 6:**
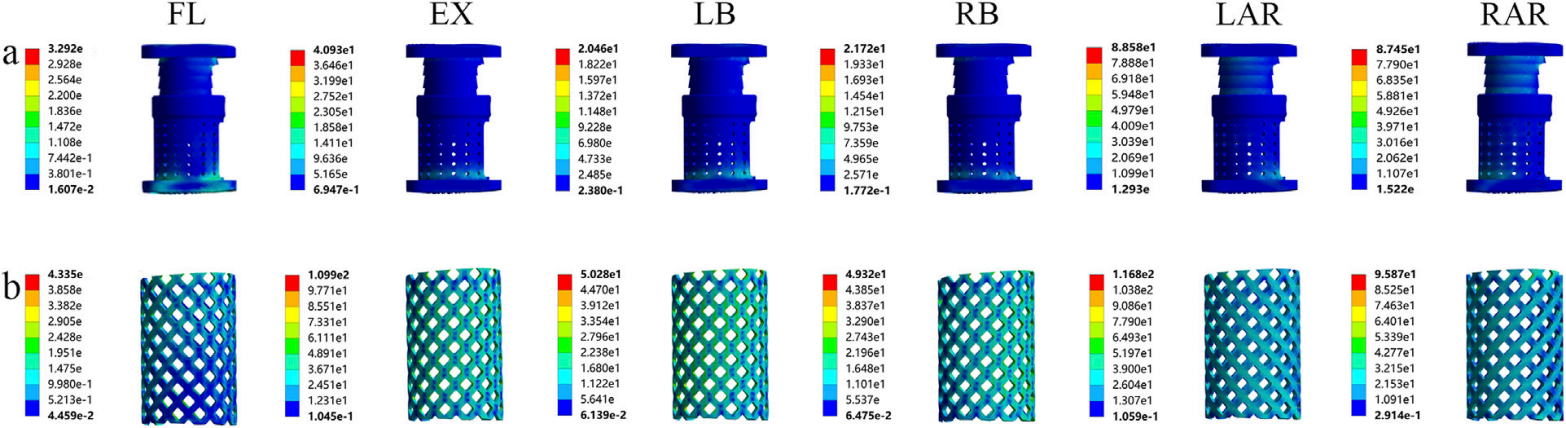
Von Mises stress of the two prostheses in different motion states: **(A)** artificial vertebral body (AVB); **(B)** titanium mesh cage (TMC) (FL: flexion, EX: extension, LB: left lateral bending, LAR: left axial rotation).

Analytical results demonstrated significant differences in peak stress distribution among the prostheses. In the AVB model, peak stresses occurred during extension (40.93 MPa) and axial rotation (88.58 MPa), followed by left and right lateral bending (20.46 MPa and 21.72 MPa, respectively), with minimal stress observed during flexion (3.92 MPa). In contrast, the TMC model exhibited 2.7-fold higher peak stress than the artificial vertebral body during extension, 1.3-fold during axial rotation, and 2.5-fold/2.3-fold during left/right lateral bending. Overall, anterior prostheses with reduced contact areas to endplates demonstrated elevated stress magnitudes. Additionally, the TMC model displayed broader stress distribution ranges but more pronounced stress concentrations (Figure 6), indicating potential failure or fracture risks under high-load kinematic conditions.

#### 3.1.4 Von mises stress distribution on the pedicle screw-rod system under different prosthetic supports

**FIGURE 7:**
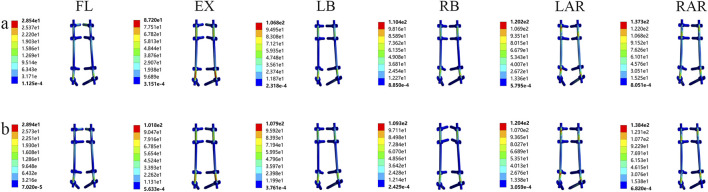
Von Mises stress of the screw-rod system supported by two kinds of prostheses: **(A)** artificial vertebral body (AVB); **(B)** titanium mesh cage (TMC) (FL: flexion, EX: extension, LB: left lateral bending, LAR: left axial rotation).

The analysis revealed no significant differences in peak stress on the posterior fixation system between different prosthetic supports (Figure 7). During extension, the von Mises stress on the TMC pedicle screw-rod system measured 101.80 MPa, approximately 1.2-fold higher than that in the artificial vertebral body group. While the TMC system exhibited marginally elevated stress levels compared to the AVB, the minimal disparity suggests that the overall mechanical performance of the posterior fixation system maintained comparable short-term stability across prosthetic support conditions. However, TMC-induced load redistribution may elevate fatigue loading during prolonged use, thereby increasing the risk of screw loosening or fracture.

#### 3.1.5 Von mises stress comparison of two models

**FIGURE 8:**
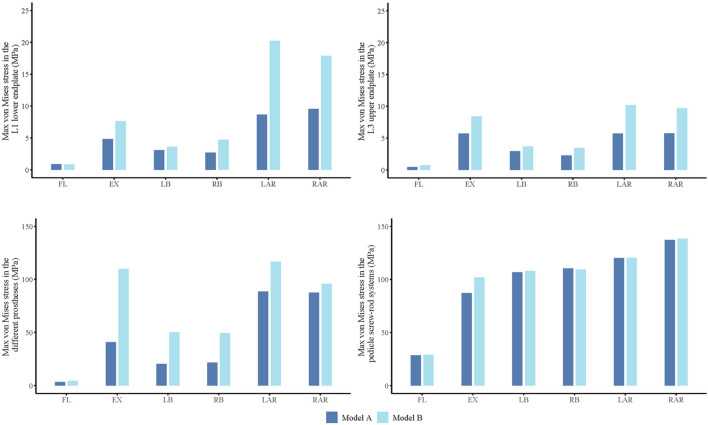
Maximum von Mises stress of L1 lower endplate, L3 upper endplate, different prostheses and screw-rod system in flexion (FL), extension (EX), left lateral bending (LB), right lateral bending (RB), left axial rotation (LAR), right axial rotation (RAR).

Finite element analysis revealed that Model B exhibited consistently higher maximum stress values at the inferior endplate of L1, the superior endplate of L3, and within the prosthesis itself compared to Model A under various loading conditions. In contrast, the maximum stress within the screw-rod system showed no substantial differences between the two models, with overall distribution patterns remaining comparable (Figure 8).

### 3.2 Clinical research

#### 3.2.1 General condition

According to the inclusion and exclusion criteria, this study enrolled 20 patients who all underwent surgical treatment and completed follow-up. Preoperative baseline demographic characteristics showed no significant intergroup differences (*p* > 0.05) (Table 2). Group A comprised 10 patients (5 males, five females) aged 30–68 years (mean 47.6 years), with lesions located in the thoracic vertebrae (*n* = 7) and lumbar vertebrae (*n* = 3), followed by 6–37 months (mean 18.1 months). Group B included 10 patients (5 males, five females) aged 25–64 years (mean 46.4 years), with thoracic (*n* = 7) and lumbar (*n* = 3) lesions, followed for 8–40 months (mean 22.1 months). Hospitalization duration was 20.10 ± 4.28 days (mean 20.1 days), operative time 351.70 ± 69.30 min (mean 357.1 min), and intraoperative blood loss 748.00 ± 458.79 mL (mean 748 mL) in Group A. Corresponding values for Group B were 18.90 ± 4.86 days (mean 18.9 days), 332.10 ± 54.48 min (mean 332.1 min), and 972.00 ± 341.43 mL (mean 972 mL). No statistically significant differences were observed between groups (*p* > 0.05).

**TABLE 2:** General information of patients in groups A and B.

Item	Group A	Group B	Test value(*t/u/χ* ^*2*^)	*P*
Age (years)	47.60 ± 14.39	46.40 ± 13.06	0.20	0.847
Male/female	4/6	5/5	0.20	0.65
Hospitalization duration	20.10 ± 4.28	18.90 ± 4.86	0.59	0.57
Operative time	351.70 ± 69.30	332.10 ± 54.48	0.70	0.49
Intraoperative blood loss	748.00 ± 458.79	972.00 ± 341.43	−1.24	0.23

#### 3.2.2 Postoperative pain recovery

Both Group A and B demonstrated significant reductions in Visual Analog Scale (VAS) scores at immediate postoperative, 1-month, 3-month, and final follow-up timepoints. No statistically significant differences in preoperative or postoperative VAS scores were observed between the two groups at immediate postoperative, 1-month, or 3-month assessments (*p* > 0.05), indicating that surgical intervention significantly alleviated pain symptoms (Table 3).

**TABLE 3:** Comparison of VAS scores between Group A and B.

Item	Preoperative	Immediate postoperative	1-month postoperative	3-month postoperative	Final follow-up
Group A	8.70 ± 0.48	6.40 ± 0.70	2.60 ± 0.52	0.80 ± 0.63	0.20 ± 0.42
Group B	8.60 ± 0.52	6.10 ± 0.74	2.20 ± 0.79	1.00 ± 0.67	0.40 ± 0.52
*U*	0.45	0.93	1.34	−0.68	−0.95
*P*	0.66	0.36	0.20	0.50	0.36

#### 3.2.3 Postoperative neurological function improvement

All 20 patients exhibited varying degrees of neurological function improvement postoperatively. ASIA scores showed no significant intergroup differences preoperatively or at immediate postoperative, 3-month, and final follow-up evaluations (*p* > 0.05) (Table 4).

**TABLE 4:** Comparison of ASIA grades between Group A and B.

Item	Grades	Preoperative	Immediate postoperative	3-month postoperative	Final follow-up
Group A	A	0	0	0	0
	B	2	1	0	0
	C	1	1	1	0
	D	7	7	1	2
	E	0	1	8	8
Group B	A	0	0	0	0
	B	3	1	0	0
	C	3	3	1	0
	D	4	5	4	3
	E	0	1	5	7
*X* ^2^		2.02	1.33	2.49	0.28
*P*		0.37	0.72	0.29	1.00

#### 3.2.4 Postoperative quality of life assessment

KPS scores significantly increased postoperatively and at the final follow-up in both groups. Preoperative and postoperative KPS scores demonstrated no statistically significant differences between groups (*p* > 0.05), confirming that surgical treatment markedly improved quality of life (Table 5).

**TABLE 5:** Comparison of KPS scores between Group A and B.

Item	Preoperative	Immediate postoperative	1-month postoperative	3-month postoperative	Final follow-up
Group A	24.00 ± 5.16	36.00 ± 5.16	50.00 ± 6.67	76.00 ± 5.16	94.00 ± 6.99
Group B	26.00 ± 5.16	33.00 ± 8.23	47.00 ± 4.83	71.00 ± 7.38	88.00 ± 6.33
*U*	−0.87	0.98	1.15	1.76	2.01
*P*	0.40	0.34	0.26	0.10	0.59

#### 3.2.5 Postoperative segmental height measurement

The study demonstrated no statistically significant differences in preoperative or immediate postoperative segmental height between Groups A and B (*p* > 0.05). However, statistically significant intergroup disparities were observed in the final follow-up segmental height and height loss (*p* < 0.05). Implant subsidence occurred in 1 case (10%) in Group A versus 7 cases (70%) in Group B, with a statistically significant difference in subsidence rates (*p* < 0.05). Group A exhibited superior outcomes compared to Group B regarding postoperative segmental height maintenance, reduced height loss, and lower subsidence rates, all showing statistical significance (*p* < 0.05) (Table 6).

**TABLE 6:** Comparison of segmental height, height loss, and subsidence rate between Group A and B.

Item	Segmental height			Height loss(mm)	Subsidence rate (%)
	Preoperative	Immediate postoperative	Final follow-up		
Group A	31.38 ± 10.37	42.13 ± 9.65	40.80 ± 9.80	1.33 ± 0.82	1/10
Group B	21.56 ± 7.81	44.07 ± 6.06	31.72 ± 8.16	12.36 ± 7.79	7/10
*T/X* ^*2*^	0.69	−0.54	2.25	−4.45	7.50
*P*	0.50	0.60	0.04	<0.01	<0.01

#### 3.2.6 Postoperative segmental angle measurement

At the final follow-up, segmental angle loss in Group A ranged from 0.21° to 2° (mean 0.77° ± 0.67°), while Group B exhibited angle loss ranging from 0.66° to 18.83° (mean 8.89° ± 6.20°). Group A demonstrated statistically significant differences compared to Group B in angle loss maintenance and kyphotic deformity correction (*p* < 0.05) (Table 7).

**TABLE 7:** Comparison of the segmental angle between Group A and B.

Item	Segmental angle			Angle loss (°)
	Preoperative	Immediate postoperative	Final follow-up	
Group A	15.18 ± 8.54	6.77 ± 5.53	7.53 ± 5.91	0.77 ± 0.67
Group B	14.97 ± 6.41	5.20 ± 3.63	14.10 ± 7.45	8.89 ± 6.20
*T*	0.06	0.75	−2.18	−4.12
*P*	0.95	0.46	0.04	<0.01

#### 3.2.7 Postoperative complications

Postoperative imaging revealed satisfactory positioning of the AVBs in all Group A patients, with no significant displacement, rotation, rod fractures, or screw loosening. In Group B, three patients exhibited marked subsidence and rotation of the titanium cage, including 1 case of rod fracture requiring revision surgery. The revision involved titanium cage replacement, long-segment dual-rod fixation, neural decompression, and spinal deformity correction, with subsequent confirmation of stable implant alignment. We selected imaging data from two representative cases: AVB reconstruction following TES (Supplementary Figure 1) and TMC reconstruction following TES (Supplementary Figure 2).

1 case of pulmonary embolism, which resolved with anticoagulant therapy; 2 cases of cerebrospinal fluid (CSF) leakage, showing improvement after antibiotic prophylaxis and fluid replacement; 1 case of pleural effusion treated with closed thoracic drainage, leading to reduced effusion volume before discharge.

## 4 Discussion

TES is a technically complex, highly risky, and extremely challenging surgical procedure designed to achieve radical tumor resection by complete removal of the pathological vertebra and surrounding involved structures, while minimizing intraoperative tumor cell dissemination and postoperative local recurrence risks (Demura et al., 2021; Kato et al., 2021; Zhang et al., 2024). As a highly specialized intervention, its success depends not only on the surgeon’s technical expertise but also on the biomechanical performance and clinical durability of vertebral reconstruction, which critically determine patients' quality of life and long-term prognosis. In this study, we innovatively integrated finite element analysis with clinical data to systematically evaluate the biomechanical characteristics and clinical efficacy of AVBs versus traditional TMCs in spinal reconstruction post-TES. The results demonstrated that AVBs exhibited significant superiority over TMCs in stress distribution uniformity, compressive stability, and long-term implant stability. These findings provide novel evidence for optimizing implant selection in TES-related vertebral reconstruction and establish a theoretical foundation for enhancing therapeutic outcomes and patients' quality of life.

Finite element analysis revealed that AVBs exhibited significantly more uniform stress distribution during load transfer, effectively dispersing mechanical loads and reducing stress concentration on adjacent vertebral endplates, thereby markedly lowering the risk of prosthetic subsidence. This finding aligns with previous studies reporting lower complication rates associated with AVBs (Wang et al., 2021c; Cao et al., 2023). Furthermore, the results substantiate the mechanical hypothesis of adjacent segment degeneration, confirming that abnormal stress concentration on adjacent endplates constitutes a key mechanism accelerating degeneration and inducing postoperative adjacent segment disease (ASD) (Radcliff et al., 2013). In contrast, TMCs, with their terminal mesh structure, demonstrate smaller contact areas with adjacent endplates. The filler material within the cages often fails to achieve complete and uniform distribution post-implantation, predisposing to localized stress concentrations. Such abnormal stress patterns not only increase endplate injury risks but may also trigger ASD. Our study indicates that while TMCs demonstrate comparable initial stability to AVBs in the early postoperative phase, their mechanical performance may progressively deteriorate under long-term loading conditions. Finite element analysis further revealed elevated and unevenly distributed stress levels in adjacent endplates following TMCs implantation. This phenomenon closely correlates with the clinical occurrence patterns of cage-related complications, providing mechanistic insights into their underlying biomechanical etiology.

Furthermore, the clinical follow-up results of this study validate the significant advantages of AVBs in spinal reconstruction following TES. Group A demonstrated superior outcomes compared to Group B in postoperative prosthetic subsidence rate, vertebral height loss, and angular loss. We suppose that these findings are closely associated with the optimized load distribution uniformity and bone-implant interface stress compatibility inherent in the artificial vertebral body design (Kiselev and Zheravin, 2024). This design characteristic not only facilitates early stabilization but also promotes long-term osseous fusion efficacy. Notably, endplate collapse and localized bone resorption were observed in some Group B cases during follow-up, suggesting that TMCs may induce complications through excessive mechanical loading on endplates (Wang et al., 2021b). Such adverse biomechanical environments may compromise fusion quality and long-term stability maintenance. The clinical significance of these findings lies in providing comprehensive and robust evidence for implant selection in post-TES spinal reconstruction. Compared with existing literature (Girolami et al., 2018; Xu et al., 2022), this study not only quantifies the biomechanical performance disparities between AVBs and TMCs under varying loading conditions through finite element modeling but also clinically verifies how these mechanical advantages translate into tangible benefits. Specifically, the AVBs' significantly reduced postoperative subsidence rate, superior anatomical adaptability, and enhanced mechanical stability provide new scientific rationale for its application in complex cases. The integrated methodology combining biomechanical modeling with clinical validation enhances the study’s credibility while offering valuable methodological references for similar research endeavors.

The optimal material selection for reconstruction following TES remains controversial. Our findings provide novel scientific evidence supporting the application of AVBs while clarifying their clinical advantages and limitations. Although AVBs demonstrate superior postoperative mechanical performance and clinical outcomes, further studies are required to evaluate their long-term efficacy and applicability across diverse clinical contexts. Existing literature extensively explores the biomechanical impacts of different vertebral reconstruction materials. Wang et al. demonstrated that the elastic modulus of materials significantly correlates with ASD risk, where both excessively high and low elastic moduli adversely affect spinal biomechanics (Wang et al., 2024). By optimizing material properties, AVBs meet load-bearing requirements while minimizing mechanical interference with adjacent structures, thereby reducing the risk of adjacent segment degeneration. Additionally, Pokorni et al. highlighted through finite element analysis that anatomical compatibility and individualized design of reconstruction materials critically enhance postoperative outcomes (Pokorni et al., 2023). This perspective aligns closely with the findings of the present study, further validating the anatomical adaptability and long-term stability advantages of AVBs.

This study systematically explored the clinical application value of AVBs in TES, while acknowledging the following limitations. Firstly, although the finite element-based biomechanical model aimed to restore anatomical structural features as comprehensively as possible, the inherent complexity of spinal dynamic loading conditions could not be fully replicated due to necessary model simplifications, particularly regarding stress distribution patterns. Secondly, the retrospective design, constrained by a limited sample size and insufficient follow-up duration, may compromise the precise assessment of long-term postoperative complications and the evolutionary trajectory of biomechanical performance. Moreover, it should be noted that this study did not perform univariate analysis on the potential impact of neoadjuvant/adjuvant chemoradiotherapy on clinical outcomes. This omission may introduce confounding factors affecting the accuracy and interpretability of our findings. To address these limitations, future research should focus on: (1) Establishing multicenter collaborative platforms to longitudinally compare clinical outcome disparities among various implantation strategies in heterogeneous patient populations through prospective cohort studies; (2) Future prospective studies should incorporate rigorous stratification or propensity score matching to investigate the independent effects of adjuvant therapies on spinal reconstruction outcomes, thereby enhancing the generalizability and clinical applicability of the conclusions; (3)Developing bone repair materials with gradient biomimetic structures that achieve dynamic equilibrium between osseointegration rate and mechanical support strength by precisely regulating material porosity and surface bioactivity; (4) Implementing personalized reconstruction protocols via radiomics technology, integrating computer-aided design to optimize prosthetic morphological parameters, thereby constructing patient-specific three-dimensional reconstruction frameworks to achieve simultaneous enhancement of biomechanical compatibility and clinical efficacy.

In conclusion, this study, through the integration of finite element analysis and clinical data review, demonstrates that AVBs exhibit significant biomechanical and clinical advantages in reconstruction following TES, providing robust evidence to guide material selection for postoperative reconstruction. Nevertheless, further research is warranted to address potential limitations and optimize their design, thereby enhancing their clinical applicability.

## 5 Conclusion

This study revealed through finite element analysis and retrospective clinical data review that AVBs reconstruction following TES demonstrates significant advantages over TMCs reconstruction in terms of uniformity of stress distribution, endplate protection, and long-term stability. Furthermore, it exhibited superior clinical efficacy in reducing postoperative subsidence, minimizing vertebral height/angular loss, and lowering complication rates.

## Data Availability

The original contributions presented in the study are included in the article/Supplementary Material, further inquiries can be directed to the corresponding authors.
