# Supplementary material for: Biomechanical and clinical comparison of different prosthetic in reconstruction following total en bloc spondylectomy in the thoracolumbar spine: based on finite element analysis and clinical data
Source: Front Bioeng Biotechnol. 2025 Jul 1;13:1573086. doi: 10.3389/fbioe.2025.1573086 (PMC12259587; doi:10.3389/fbioe.2025.1573086)
Supplement: Supplementary file 1 [file DataSheet1.pdf]

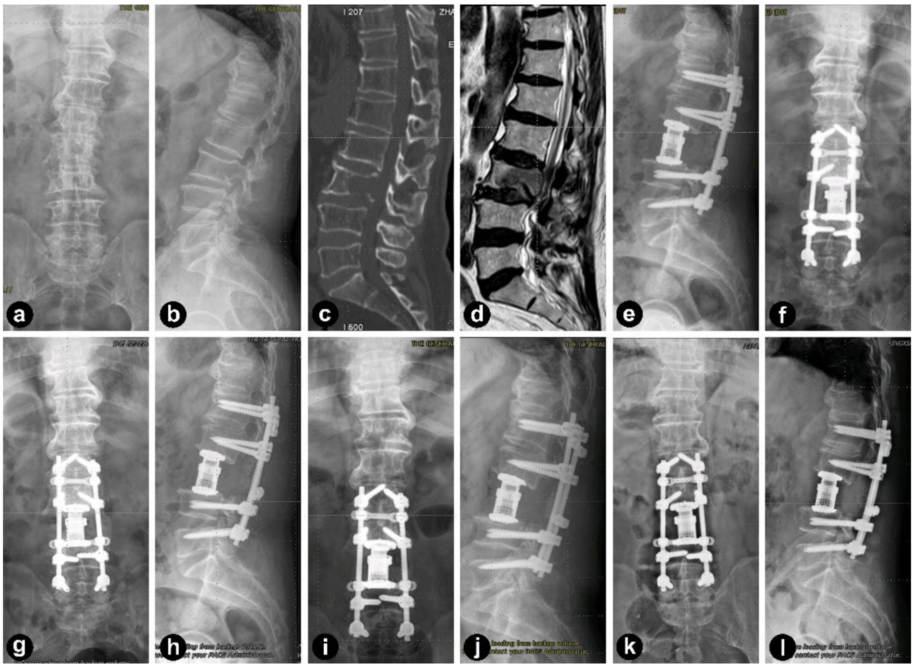

Supplementary Figure 1. Imaging of L3 vertebral plasmacytoma treated with AVB reconstruction: a-d. Preoperative lumbar spine X-rays (anteroposterior and lateral views), CT, and MRI sagittal views; e, f. Immediate postoperative lumbar spine X-rays (AP and lateral); g, h. 1-month postoperative lumbar spine X-rays (AP and lateral); i, j. 3-month postoperative lumbar spine X-rays (AP and lateral); k, l. 1-year postoperative lumbar spine X-rays (AP and lateral).

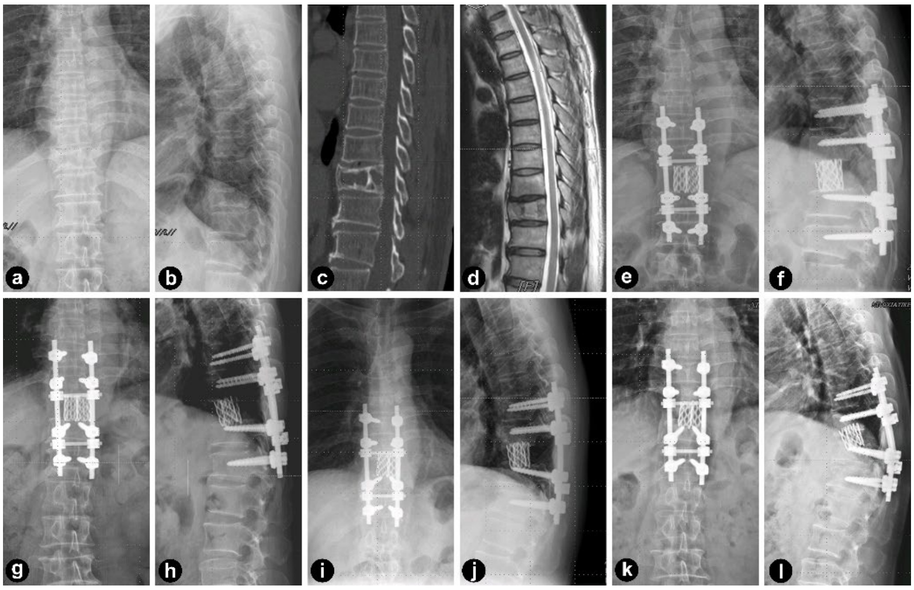

Supplementary Figure 2. Imaging of instrumentation failure in T10 metastatic bone tumor reconstruction with TMC: a-d. Preoperative thoracic spine X-rays (anteroposterior and lateral views), CT, and MRI sagittal views; e, f. Immediate postoperative thoracic spine X-rays (AP and lateral); g, h. 1-month postoperative thoracic spine X-rays (AP and lateral); i, j. 1-year postoperative thoracic spine X-rays (AP and lateral); k, l. 2-year postoperative thoracic spine X-rays (AP and lateral).
